# Vibrational Properties of *h*-BN and *h*-BN-Graphene Heterostructures Probed by Inelastic Electron Tunneling Spectroscopy

**DOI:** 10.1038/srep16642

**Published:** 2015-11-13

**Authors:** Suyong Jung, Minkyu Park, Jaesung Park, Tae-Young Jeong, Ho-Jong Kim, Kenji Watanabe, Takashi Taniguchi, Dong Han Ha, Chanyong Hwang, Yong-Sung Kim

**Affiliations:** 1 Korea Research Institute of Standards and Science, Daejeon 305-340, Korea; 2 University of Science and Technology, Daejeon 305-350, Korea; 3 Department of Physics, ChungNam National University, Daejeon 305-764, Korea; 4 Department of Physics, Yonsei University, Seoul 120-749, Korea; 5 Advanced Materials Laboratory, National Institute for Materials Science, 1-1 Namiki, Tsukuba 305-0044, Japan

## Abstract

Inelastic electron tunneling spectroscopy is a powerful technique for investigating lattice dynamics of nanoscale systems including graphene and small molecules, but establishing a stable tunnel junction is considered as a major hurdle in expanding the scope of tunneling experiments. Hexagonal boron nitride is a pivotal component in two-dimensional Van der Waals heterostructures as a high-quality insulating material due to its large energy gap and chemical-mechanical stability. Here we present planar graphene/*h*-BN-heterostructure tunneling devices utilizing thin *h*-BN as a tunneling insulator. With much improved *h*-BN-tunneling-junction stability, we are able to probe all possible phonon modes of *h*-BN and graphite/graphene at *Γ* and *K* high symmetry points by inelastic tunneling spectroscopy. Additionally, we observe that low-frequency out-of-plane vibrations of *h*-BN and graphene lattices are significantly modified at heterostructure interfaces. Equipped with an external back gate, we can also detect high-order coupling phenomena between phonons and plasmons, demonstrating that *h*-BN-based tunneling device is a wonderful playground for investigating electron-phonon couplings in low-dimensional systems.

Investigating phonons, the fundamental vibrational modes of a crystal lattice, is important to understand many physical mechanisms such as heat capacity, thermal and electrical conductivities. Especially in low-dimensional systems such as graphene- and carbon nanotube-based devices, electron-phonon scattering has been considered as the major source for limiting conductivity^1^,^2^. Along with other experimental methods to probe phonon excitations such as neutron scattering^3^, x-ray scattering^4^ and electron energy-loss spectroscopy^5^, Raman spectroscopy has been widely used to characterize lattice motions because of its easy-and-quick application and high sensitivity even for nanoscale devices^6^. However, Raman-active phonons are limited to a few vibration modes due to stringent optical selection rules. Inelastic electron tunneling spectroscopy (IETS) has proven its capability in probing phonon excitations without selection rule limitations^7^,^8^,^9^. Initial IETS studies on graphene devices with scanning tunneling microscope (STM) measurements, however, have detected no more than one or two phonon excitations because of weak IETS signals and the limited stability of tunneling junction composed of the STM probe, vacuum barrier and graphene devices^10^. Recently, Natterer *et al*. reported that up to six phonon excitations of graphene can be observed by improving IETS signal/noise ratio in STM measurements^11^.

Hexagonal-boron nitride (*h*-BN) has been acclaimed as a superb gate-insulating material for low-dimensional electronic and optical devices with graphene^12^,^13^, single-walled carbon nanotubes^14^ and transition metal dichalcogenides (TMDC)^15^ enabling the engineering of low-dimensional platforms for novel electronic properties such as Hofstadter butterfly effects^16^,^17^,^18^. Thus, investigating phonons of *h*-BN and its hybrid structures are greatly needed, not only for understanding physical properties of *h*-BN-based nanoscale devices but also for developing new electronic and optical devices. Although there are several experimental and theoretical reports on *h*-BN phonons^19^,^20^,^21^,^22^,^23^,^24^,^25^, discussions on phonon excitations of *h*-BN-based heterostructures are sparse with a few of experimental reports on Raman-active *E*_2g_ phonons^26^ and theoretical expectations from the first principle calculations^27^. Moreover, most of experimental reports on *h*-BN relied on optical methods due to a large band gap (≈6 eV) of *h*-BN^24^, which makes IETS studies with STM challenging.

In this article, we present electron-tunneling spectroscopy measurements of gated single-layer graphene devices with a thin *h*-BN layer as a tunneling insulator^28^,^29^,^30^,^31^. By adapting a conventional planar-tunneling scheme, we establish a stable tunneling junction with high-quality *h*-BN, which is less sensitive to experimental noises such as mechanical vibrations, improving IETS signal/noise ratio in a significant manner. We are able to measure twelve phonon excitations below 200 meV, which we assign to the phonons of *h*-BN, graphite and graphene/*h*-BN heterostructure (Table 1). In our tunneling devices, electrons tunnel through thin (3 or 4 layers) *h*-BN insulator, which makes it possible to detect *h*-BN phonons by IETS for the first time. We observe all possible phonon excitations of *h*-BN at the high symmetry points of ***Γ*** and ***K*** in the Brillouin zone. Quite surprisingly, the majority of phonon excitations at the symmetry point ***M*** are missing, opposite to the previous IETS studies with STM measurements^11^,^32^, suggesting that tunneling electrons in our *h*-BN planar junctions obey parallel-momentum conservation and would be less susceptible to momentum-changing scattering events. When graphene and *h*-BN layers are brought together forming a graphene/*h*-BN heterostructure, it is found that low-frequency out-of-plane motions of *h*-BN and graphene lattices are significantly altered at the graphene/h-BN interface: ZA modes of both *h*-BN and graphene phonons become hardened. We calculate phonon dispersions of *h*-BN, graphite and graphene/*h*-BN heterostructure with density functional perturbation theory (DFPT) finding good agreement with experimental data. Finally, we are able to identify charge-density-dependent excitations with energies higher than 200 meV, suggesting that the overtone excitations can be due to multi-phonon scattering processes combined with charge-density-dependent excitations, plasmons^8^.

The inset of Fig. 1a displays the schematic of our *h*-BN-based tunneling devices with both thick *h*-BN (>20 nm) and SiO_2_ (300 nm) as gate insulators, and a doped Si substrate as bottom gate. Sample bias (*V*_b_) is applied to the top probe, and tunneling current as well as differential conductance (*G* = d*I*/d*V*_b_) are monitored through the graphene layer. We use a thick-and-narrow graphite flake as the tunneling probe, which shows superior tunneling characteristics to metal electrodes^33^,^34^,^35^. We ensure that tunneling junctions remain clean by sequentially employing a dry-transfer method and high-temperature annealing^12^ (Methods).

Figure 1a–c show tunneling spectra curves from one of single-layer graphene devices at *T* = 5.9 K. We apply small AC-excitation voltage (*V*_rms_ = 1 mV, *f* = 433. 3 Hz) to DC sample bias and measure both *I*-*V*_b_ (Fig. 1a) and *G* = d*I*/d*V*_b_ (Fig. 1b). In addition, the conductance derivative, d*G*/d*V*_b_ = d^2^*I*/d*V*_b_^2^, directly proportional to the IETS signal can be measured simultaneously by another lock-in synchronized at the frequency of 2*f*. The most prominent structures in d*I*/d*V*_b_ spectrum (Fig. 1b) are the dip in the vicinity of *V*_b_ = −300 mV where the charge neutrality point (*E*_D_) of graphene is expected to be at gate voltage *V*_g_ = −40 V and the dip at the Fermi level, *E*_F_ (*V*_b_ = 0 mV). Additionally, we can find several kinks in d*I*/d*V*_b_ around *E*_F_ within |*V*_b_| ≤ 200 mV. These features become more prominent in the IETS signal d*G*/d*V*_b_ (Fig. 1c), appearing as peaks and dips at different polarities of *V*_b_.

To analyze spectrum features in detail, we obtain a series of tunneling spectra while varying the charge density with *V*_g_. Figure 1c shows a sequence of d*G*/d*V*_b_ curves at gate voltages ranging from *V*_g_ = −50 V to −40 V (lower part, hole doping) and *V*_g_ = 50 V to 40 V (upper part, electron doping) with a spacing of Δ*V*_g_ = 2 V. Without any difficulties, we can identify that there exists two types of tunneling features, with and without density dependence. As expected, the feature related to *E*_D_ changes its position upon varying charge density, as marked with red dotted arrows. However, most spectra features within |*V*_b_| ≤ 200 mV barely change their positions in sample bias with a little variation in intensity even though charged carriers switch polarity from electrons to holes in the full range of gate voltages, −50 V ≤ *V*_g_ ≤50 V.

More details of the tunneling spectra can be seen in the gate mappings. As proven in the previous measurements^36^,^37^,^38^, the two-dimensional display of tunneling spectra as functions of *V*_g_ and *V*_b_ reveals a plethora of electronic structures not just limited to *E*_F_ but also extended to both empty (*V*_b_ >0 mV) and filled (*V*_b_ <0 mV) states. In the |d*G*/d*V*_b_| gate mapping (Fig. 2a), the position of *E*_D_ can be easily identified as the dark-oblique line running diagonally across the map. We point out that the position of *E*_D_ in our tunneling devices is determined by not only the displacement fields through *h*-BN tunneling and back-gate insulators but also by quantum capacitance of graphene^31^. However, detailed analysis of this subject is out of scope for the current report and will be presented elsewhere.

We now focus on the inelastic tunneling features, which do not show dependence on *V*_g_. As seen in Fig. 1c, most features in d*G*/d*V*_b_ barely move in *V*_b_ while varying *V*_g_, which can be further confirmed in the mappings of Fig. 2a,b as structures running parallel to the axis of *V*_g_. It is well known that the tunneling current is enhanced by inelastic electron tunneling effects which form additional tunneling channel at the threshold energy for respective excitation^39^. Thus, signals related to IETS can be identified as symmetric kinks in *G* = d*I*/d*V*_b_ (Fig. 1b), and peaks and dips in the second derivative of the tunneling spectra, d*G*/d*V*_b_ = d^2^*I*/d*V*_b_^2^ (Fig. 1c)^8^,^32^. In Fig. 2a, we intentionally convert dips to peaks by taking absolute values in |d*G*/d*V*_b_| to highlight the symmetry of IETS features around the *E*_F_. Figure 2b is the magnified image of the dotted-white box region in Fig. 2a. In total, we are able to identify twelve independent IETS features within the window of |*V*_b_| ≤ 200 mV, which we ascribe to the inelastic tunneling signals due to the phonons of *h*-BN, graphite and graphene/*h*-BN heterostructure.

Averaging out individual d*G*/d*V*_b_ spectra at different *V*_g_ (Fig. 2a) proves to be quite useful to identify IETS excitations^11^ since gate-voltage independent IETS signals remain as either strong peaks or dips but gate-voltage dependent features such as those relating to *E*_D_ become diminished. To analyze phonon modes more accurately, however, we choose the area where gate-dependent features are less conspicuous as shown in Fig. 2b. The black circles in Fig. 2c are the averaged d*G*/d*V*_b_ curve from 101-independent d*G*/d*V*_b_ spectrum in the gate voltage range from *V*_g_ = −50 V to *V*_g_ = −10 V with the spacing of Δ*V*_g_ = 0.4 V. To illustrate the symmetry of the phonon-related IETS signals, averaged d*G*/d*V*_b_ spectra in the filled (*V*_b_ < 0 mV) state obtained from *V*_g_ = 10 V to *V*_g_ = 50 V are also plotted (blue circles in Fig. 2c). We extract the positions and FWHMs of twelve excitations with multi-peak Lorentzian fitting and the overlaid red line in Fig. 2c is the direct summation of twelve independent Lorentzian graphs (brown curves).

First, we compare our data with DFPT-calculated phonon dispersions of 4-layer *h*-BN (Fig. 3a), which has a few of characteristic phonon modes distinctive from graphite and graphene phonons (Fig. 3b). As indicated in Fig. 3a, we can identify all *h*-BN phonons at and near high symmetry points ***Γ*** and ***K***. For example, the strongest peak in tunneling spectra at ≈168 mV (*P*_10_ in Fig. 2) is attributed to the in-plane optical ***Γ***_**5+**_ phonon mode (LO/TO) of *h*-BN, which is Raman-active *E*_2g_ phonon at the frequency of ≈1365 cm^−1^ (≈169 meV)^19^,^40^. Additionally, out-of-plane optical ***Γ***_**3−**_ (or ***Γ***_**4+**_) (ZO) and acoustic ***Γ***_**4+**_ (ZA) phonon modes can be assigned to the peaks at *P*_6_ (≈103 meV) and *P*_1_ (≈17 meV), respectively. We can argue that ***Γ***_**4+**_ phonon mode (ZA, *P*_1_) becomes hardened because 4-layer *h*-BN is encapsulated with relatively thick top graphite probe and bottom *h*-BN gate insulators. We can recognize several ***K***-point phonon modes as well: in-plane optical ***K***_**1,2**_ (LO, *P*_9_) and ***K***_**5**_ (TO, *P*_8_), in-plane acoustic ***K***_**5**_ (LA, *P*_7_) and ***K***_**1,2**_ (TA, *P*_6_), and out-of-plane optical ***K***_**6**_ (ZO, *P*_4_) and acoustic ***K***_**6**_ (ZA, *P*_2_) phonon modes. Note that some of phonon modes especially out-of-plane acoustic ***K***_**6**_ (ZA) phonons are slightly off from theoretical expectations, which we relate to graphene/h-BN heterostructures. We will discuss how these phonon modes are influenced by nearby layers later. In addition, we tend to assign the peak at ≈186 mV (≈1500 cm^−1^, *P*_11_) to the overbending mode of the in-plane optical (LO, *S*_1_, *T*_3_) phonons of *h*-BN, as marked with a red star in Fig. 3a. Reich *et al*.^19^ observed similar LO-overbending effects from bulk *h*-BN crystals at the frequency of 1470 cm^−1^ (≈182 mV) with resonant Raman measurements, and Kern *et al*.^21^ theoretically argued that the highest point (≈1490 cm^−1^, 185 mV) of LO-phonon branch is formed not at the high symmetry point of ***Γ***but in between ***Γ*** and ***K*** resulting LO-overbending branch.

As mentioned previously, we employed graphite flake as a tunneling probe, therefore we can expect that our data should have signatures of graphite phonons. Indeed, we can identify several graphite phonons at the high symmetry points of ***Γ*** and ***K***: in-plane optical ***Γ***_**5+**_ (LO/TO, *P*_12_) and out-of-plane optical ***Γ***_**4+**_ (ZO, *P*_6_) phonon modes, and in-plane optical ***K***_**1,2**_ (TO, *P*_10_) , ***K***_**5**_ (TO, *P*_9_), and in-plane acoustic ***K***_**2**_ (TA, *P*_7_) phonon modes along with out-of-plane optical/acoustic ***K***_**6**_ (ZO/ZA, *P*_4_) phonon modes. It is worth mentioning that phonon dispersions for graphite and single-layer graphene are almost identical (Fig. 3b). Thus, we could assign above-mentioned excitations to graphene phonons as well, except the out-of-plane acoustic ***Γ***_**6−**_ (or ***Γ***_**2−**_) (ZA, *P*_1_) phonon mode which is solely ascribed to the layered structure of graphite. Note that several excitations could have contributions from both *h*-BN and graphite/graphene phonons because of the structural similarity of *h*-BN and graphite/graphene. However, the peak at ≈199 mV (*P*_12_) are exclusively from the ***Γ***_**5+**_phonon mode of graphite/graphene, which is Raman-active *E*_2g_ phonon (G peak) at ≈1580 cm^−1^ (≈196 meV)^6^, and LO-overbending (≈202 mV) around ***Γ*** point^32^.

It is quite surprising that the majority of phonon excitations of graphite/graphene at the symmetry point ***M*** are missing differing from the previous IETS studies with STM tunneling measurements^10^,^11^,^32^, except the ***M***_**2+**_ phonon (ZO, *P*_5_) mode. For example, ***M***_**3−**_ phonon (TO, ≈177 meV) mode of graphite/graphene marked with a black star in Fig. 3b is considered as the most populated phonon mode (Fig. S1), but no sign of ***M***_**3−**_ phonons are observed. Additionally, ***M***_**3+**_ phonon (ZA, ≈58 meV) mode, one of the conspicuous phonons with large density-of-states (DOS), is missing in our IETS measurements. ***M***-phonon suppression is observed in *h*-BN phonons as well. Based on DFPT calculation (Fig. S1) and other previous reports^20^,^21^,^41^, the phonon mode with the largest DOS is from the TO branch connecting ***K*** (***K***_**1**_, ***K***_**2**_) and ***M*** (***M***_**2+**_) points, which could be linked to the excitation at ≈156 meV (*P*_9_). However, the strongest IETS signal in our devices is from the ***Γ***_**5+**_ phonon mode at ≈168meV, which has considerably lower phonon DOS compared to other ***M***-point phonons (Fig. S1). In addition, we can argue that ***M***_**2+**_ (or ***M***_**3−**_) phonon (TA) mode at ≈68 meV is also missing in the tunneling spectra given the fact that the excitation at ≈67 meV (*P*_4_) is the weakest IETS feature (Fig. 2c), which could be solely ascribed to the ***K***_**6**_ phonon modes of *h*-BN and graphite/graphene.

In both graphene and graphite, all available electronic states of tunneling electrons are located in the vicinity of the ***K*** point in the Brillouin zone. Thus, significant amount of momentum relaxation should be required in order for electrons to interact with or to be scattered by the phonons at ***M*** point. On the contrary, phonons at the ***Γ*** point can scatter electrons without changing crystal momentums (intra-valley scattering). Even though electrons require momentum change (inter-valley scattering) to interact with phonons at the ***K*** point, the electron-phonon scattering rate for the phonons at ***K*** point could be higher than the case for ***M***-point phonons, which require multiple electron-phonon scatterings. Note that the excitation at ≈84 meV (*P*_5_), which we tentatively assign to the ***M***_**2+**_ phonon (ZO) mode, is one of the broadest excitations among twelve peaks with ≈18 mV FWHM. Here, we conclude that the tunneling in our planar *h*-BN junctions would be less susceptible to momentum-changing scattering events, especially parallel momentums during tunneling process, contrast with STM measurements in which one electrode is an atomically sharp non-planar metal tip, which breaks parallel-momentum conservation^7^,^10^,^11^,^32^.

When graphene and *h*-BN layers form heterostructure, it is expected that the lattice dynamics of graphene and *h*-BN, especially low-frequency out-of-plane modes, are influenced by the nearby layers. Indeed, we find that graphene/*h*-BN heterostructure shows phonon dispersions different from those of free-standing graphene and *h*-BN. Figure 3c displays the DFPT-calculated phonon dispersions of graphene/*h*-BN heterostructure. For the sake of simplicity, we consider single-layer graphene is aligned on top of *h*-BN layer in an AA stacking, with graphene atoms (unit length: 0.245 nm) relaxed to *h*-BN lattice (unit length: 0.251 nm). To compensate the lattice deformation (strains) due to the structural mismatch, we calculate Grüneisen parameters for free-standing graphene and *h*-BN, and obtain the phonon modes of the unstrained graphene/*h*-BN heterostructure (Fig. 3c) (Methods). First, we notice that out-of-plane acoustic-phonon branch of *h*-BN becomes hardened shifting ***K***_**6**_ phonon (ZA) mode to higher frequency (≈46 meV), which can be assigned to the previously-unassigned peak at ≈47 mV (*P*_3_). Second, out-of-plane acoustic ***Γ***_**6−**_(or ***Γ***_**2−**_) phonon (ZA) mode of graphene is blue-shifted to a frequency of ≈36 meV (*P*_2_). Third, ***M***_**2+**_phonons (ZO) of graphene become hardened, shifting closer to the experimental data at ≈86 meV (*P*_5_). Last, graphene LA-phonon branch becomes slightly softened, moving ***K***_**5**_ phonons closer to the peak at ≈140 meV (*P*_8_).

We also calculate phonon dispersions of AB-stacked graphene/*h*-BN heterostructure, which reveal out-of-plane motions of graphene and *h*-BN lattices are affected less than those at the AA-stacked interface (Fig. S2). It is safe to assume, however, that both AA- and AB-stacked graphene/*h*-BN junctions coexist in our tunneling junctions, where active tunneling area can be as large as the area of the graphite probe which is up to several microns. Table 1 lists a summary of observed IETS excitations with the most plausible phonons from *h*-BN, graphite and graphene/*h*-BN heterostructure based on the available phonon DOS. In the table, we also list the DFPT-calculated phonon energies compared with other theoretical expectations^21^ and experimental observations^4^,^19^,^32^, which show good agreement with our data.

We now discuss charge-density-dependent IETS spectra. As indicated with dotted-yellow boxes in Fig. 2a and displayed in magnified d*G*/d*V*_b_ mappings in Fig. 4, we can identify at least four (three) excitations change positions in positive (negative) *V*_b_ as *V*_g_ varies, differing from phonons that do not change their position in energy. Thus, we can argue that observed charge-density-dependent IETS features could be due to multilevel scattering processes involved with charge-density-independent phonons and charge-density-dependent excitations such as plasmons. To extract the baseline-phonon excitation, we use an exponential fit with *V*_b,i_ (*V*_g_) = *E*_i_ + *α* exp^(*β V*_g_), and find *E*_1_ ≈ 214 mV (*E*_2_^*^ ≈ −201 mV) for the empty (filled) state excitation in Fig. 4a,b. Other red-dashed lines at lower energies are fitted to the data using the same fitting parameters **a** and **b**, obtaining *E*_2_ ≈ 200 mV, *E*_3_ ≈ 181 mV (*E*_3_^*^ ≈ −183 mV) and *E*_4_ ≈ 167 mV (*E*_4_^*^ ≈ −170 mV) in positive (negative) *V*_b_. As displayed in Fig. 4, excellent fits suggest that the charge-density-dependent excitations, which are unclear at this moment, should be the same for all observed features. Note that some of the baseline excitations such as *E*_1_, *E*_2_ and *E*_2_^*^ are higher than the highest phonon energy of our systems (≈200 mV, graphite ***Γ***_**5+**_phonons), suggesting that these overtone modes could be due to multi-phonon scattering events. Interestingly, we are able to find the combinations of two-phonon modes, comparable to each of the baseline excitation: *E*_1_ with *P*_3_ + *P*_10_ (≈215 mV), *E*_2_ (*E*_2_^*^) with *P*_3_ + *P*_9_ (≈204 mV), *E*_3_ (*E*_3_^*^) with *P*_3_ + *P*_8_ (≈183 mV), and *E*_4_ (*E*_4_^*^) with *P*_3_ + *P*_7_ (≈174 mV) sharing *P*_3_, out-of-plane acoustic ***K***_**6**_ phonons of *h*-BN as a common excitation.

In summary, we have performed inelastic electron tunneling spectroscopy studies of graphene/*h*-BN heterostructures with a thin *h*-BN layer as a tunneling insulator and observed all the available phonon modes of *h*-BN at the ***Γ***and***K*** high symmetry points by electrical transport measurements for the first time. We find out out-of-plane motions of graphene and *h*-BN lattices become hardened when graphene/*h*-BN heterostructure is formed. Additionally, we observe charge-density-dependent excitations, which could be related to both multi-phonon and plasmon-related scattering processes. With IETS employing *h*-BN as a tunneling insulator, we have demonstrated that *h*-BN-based tunneling devices become a powerful experimental playground for exploring not only novel electronic structure^28^,^30^,^42^ but also various types of collective excitations in graphene/*h*-BN hybrid devices as well as other low-dimensional materials such as TMDCs and carbon nanotubes. Moreover, benefited by much-improved-stable tunneling junctions, our tunneling devices make it possible to probe electronic and mechanical properties of nanoscale devices with applications of external variants such as magnetic fields, electro-magnetic waves, temperature, and pressure where previous tunneling experiments have been limited.

## Methods

### Device Fabrication

Graphene/*h*-BN tunneling devices are fabricated using multiple steps of dry-transfer method developed by Dean *et al*.^12^. At first, bottom thick *h*-BN (>20 nm) are mechanically exfoliated from high-quality single crystals and transferred on thermally grown 300 nm thick SiO_2_ on Si. Next, single-layer graphene, thin *h*-BN (3 or 4 layers) and graphite flakes are sequentially transferred on top of bottom *h*-BN insulator. For each step, we first prepare Si substrates spin coated with water-soluble Poly-StyrenSulfonic (PSS) acid and PMMA. Desirable flakes mechanically exfoliated on the polymer-stacked Si substrates are examined under an optical microscope and later transferred to pre-defined locations with micro-manipulating transfer stage. Before dissolving PMMA in acetone, we bake out the samples at 200 °C for 2 minutes to promote the adhesion between transferred layers. It is critically important to have clean and flat interfaces for high-quality tunneling devices. To further remove any polymer residues and reduce the number of bubbles formed in 2-D heterostructures, we anneal the samples in the mixture of Ar and H_2_ (9:1 ratio by flow rate) at the elevated temperature 350 °C for more than 4 hours. In total, we applied dry-transfer method three times and high-temperature annealing four times before loading the samples in the cryostat for measurements. Additional annealing process is performed after fabricating Ti/Au (5 nm/55 nm) electrodes with electron beam lithography.

### Numerical Calculation

**Figure 3:**
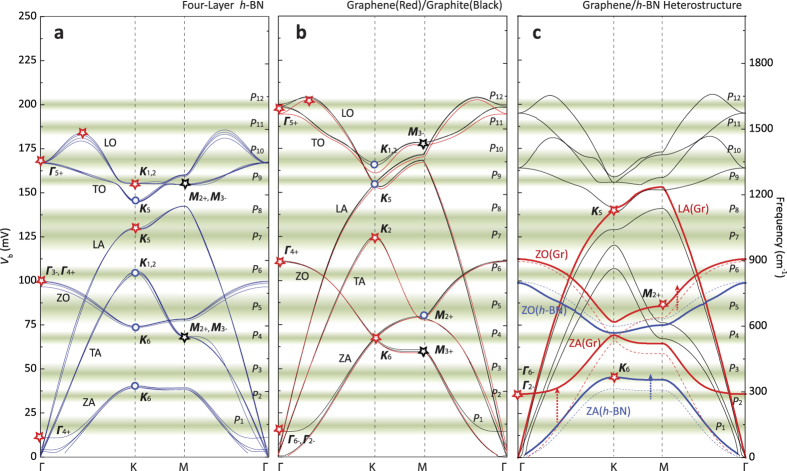
Phonon dispersions of *h*-BN, graphite/graphene and graphene/*h*-BN heterostructure. (**a**) DFPT-calculated phonon dispersions of *h*-BN (4 layers) (**b**) graphite/graphene and (**c**) AA-stacked graphene/*h*-BN heterostructure. The dashed blue and red lines in Fig. 3c are phonon dispersions of out-of-plane optical and acoustic branches of free-standing single-layer *h*-BN and graphene, respectively, for comparison. The positions and widths of green-shaded strips represent energy and FWHM of each IETS peak in Fig. 2. Red stars indicate the most plausible phonon modes seen in our data, blue circles are other available phonon modes at high symmetry points (Table 1). Black stars in Fig. 3a,b mark the ***M***-point phonons which are not seen in our data (Fig. 2). Each phonon mode is labeled with notations based on the symmetry group.

Phonon dispersions of *h*-BN, graphite/graphene and graphene/*h*-BN heterostructures are calculated using density functional perturbation theory (DFPT)^43^,^44^ implemented in the Vienna Ab initio Simulation Package (VASP)^45^ within local density approximation (LDA)^46^. The projector augmented pseudopotentials (PAW)^47^ are used and the plane-wave cutoff in the plane-wave expansion is 500 eV. We use 6 × 6 × 1 supercell for calculation. The calculated equilibrium lattice constants for graphene and *h*-BN are a_GR_ = 0.245 nm and a_*h*-BN_ = 0.251 nm, respectively, which are close to the experimentally observed values within 1%^1^,^24^. In order to calculate phonon dispersions of graphene/*h*-BN heterostructure, we use the 6 × 6 × 2 supercell, assuming that single-layer graphene is commensurately aligned on top of *h*-BN layer in an AA-stacking direction. With obtained Grüneisen parameters for free-standing graphene (γ_GR_) and *h*-BN (γ_*h*-BN_) layer, we calculate the phonon modes of the unstrained graphene/*h*-BN heterostructure, as shown in Fig. 3c. For comparison, in the compressive-strained-*h*-BN/graphene heterostructure (a_GR_ = 0.245 nm), the phonon modes of *h*-BN are increased by <γ_*h*-BN_ > = 1.13% in average compared with the unstrained graphene/*h*-BN heterostructure. Additionally, for the tensile-strained-graphene/*h*-BN heterostructure (a_BN_ = 0.251 nm), the phonon modes of graphene is decreased by <γ_GR_> = 1.43% in average, with respect to the unstrained graphene/*h*-BN heterostructure. Although AB-stacked graphene/h-BN heterostructure is expected to be more stable than its counterpart AA-stacked heterostructure^27^, the calculated phonon dispersions of the AA-stacked heterostructure are found to be in better agreement with our observations. However, we can safely assume that both AA-stacked and AB-stacked graphene/h-BN heterojunctions exist in our tunneling junction since our graphene/*h*-BN heterostructures are fabricated using dry-transfer method^12^ without additional efforts on the alignment of crystalline direction with graphene and *h*-BN lattices, resulting in the atomic-stacking sequence is arbitrary.

## Additional Information

**How to cite this article**: Jung, S. *et al*. Vibrational Properties of h-BN and h-BN-Graphene Heterostructures Probed by Inelastic Electron Tunneling Spectroscopy. *Sci. Rep*. **5**, 16642; doi: 10.1038/srep16642 (2015).

## Supplementary Material

Supplementary Information

## Figures and Tables

**Figure 1:**
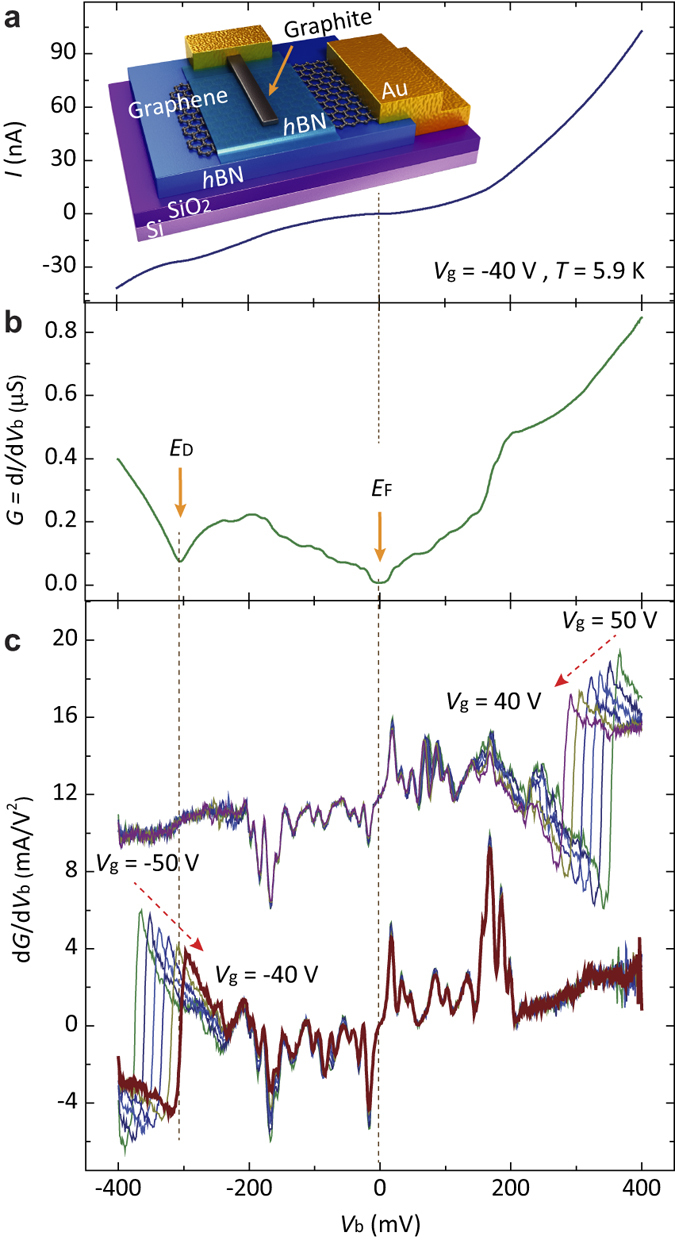
Tunneling spectra with thin *h*-BN as a tunneling insulator. (**a**) (inset) Schematic of our tunneling devices utilizing thin *h*-BN as a tunneling insulator. Back-gate voltage (*V*_g_) is applied through Si/(300 nm)SiO_2_ substrate and thick (>20 nm) *h*-BN layers. Sample bias (*V*_b_) is applied to the top-graphite probe and tunneling current is monitored through bottom graphene. Current-voltage (*I*-*V*_b_) tunneling spectrum measured at *V*_g_ = −40 V and *T* = 5.9 K. (**b**) Differential conductance (*G* = d*I*/d*V*_b_) curve measured with an AC-excitation voltage, *V*_rms_ = 1 mV at *f* = 433.3 Hz. Tunneling spectra presented in Fig. 1a,b are measured simultaneously. Orange arrows indicate the Fermi level (*E*_F_) and the Dirac point (*E*_D_) at *V*_g_ = −40 V. (**c**) Conductance derivative (d*G*/d*V*_b_) numerically obtained from the spectrum presented in Fig. 1b (brown-bold curve). d*G*/d*V*_b_ spectra as a function of *V*_g_ in steps of Δ*V*_g_ = 2 V in hole-doped (lower part) and electron-doped (upper part) regions. IETS signals are seen as peaks and dips, positioned symmetrically around *E*_F_.

**Figure 2:**
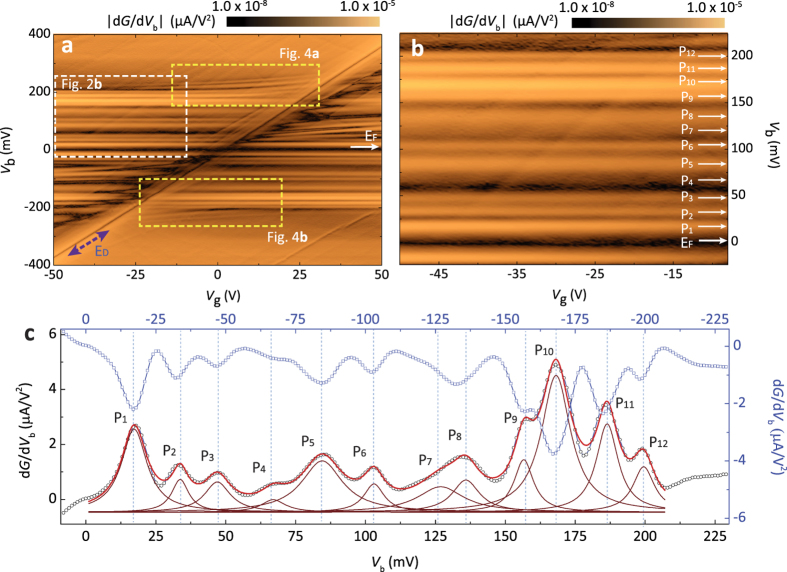
d*G*/d*V*_b_ spectra featuring IETS phonon excitations. (**a**) High resolution |d*G*/d*V*_b_| gate mapping at *T* = 5.9 K varying *V*_g_ from *V*_g_ = −50 V to *V*_g_ = 50 V with a step of Δ*V*_g_ = 0.4 V. The positions of *E*_F_ and *E*_D_ are marked with white and dark-blue arrows, respectively. We take the absolute values in d*G*/d*V*_b_ to highlight the symmetric IETS features around *E*_F_. (**b**), Magnified image of the dotted-white box region in Fig. 2a. Each parallel line marked with a white arrow represents individual IETS excitation. (**c**) Averaged d*G*/d*V*_b_ curve from 101-individual tunneling spectra in Fig. 2a from *V*_g_ = −50 V to *V*_g_ = −10 V (black circles). Red overlaid line and brown curves are from multi-peak Lorentzian fittings without baseline subtraction. The overhanging curve (blue circles) is the averaged d*G*/d*V*_b_ spectra from *V*_g_ = 10 V to *V*_g_ = 50 V.

**Figure 4:**
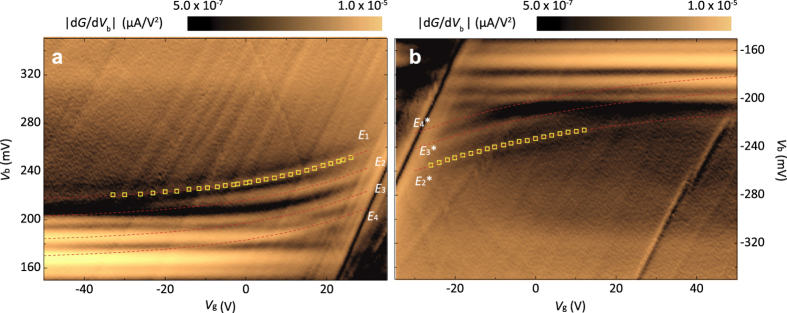
IETS spectra dependent on charge density. (**a**,**b)** Magnified d*G*/d*V*_b_ gate-mapping images from the dotted-yellow box areas in Fig. 2a for the empty state (*V*_b_ >0 mV, **a**) and the filled state (*V*_b_ < 0 mV, **b**). Yellow squares mark the peak positions of the highest overtone excitation at different *V*_g_ and red-dotted lines are from the numerical fitting based on the peak positions for *E*_1_ (**a**) and *E*_2_* (**b**), respectively. Other red-dotted lines for *E*_2_, *E*_3_ (*E*_3_*) and *E*_4_ (*E*_4_*) are fitted to the data as described in the text.

**Table 1:** Phonon energies obtained from IETS spectra with thin *h*-BN as a tunneling insulator.

Peak No.	Position (mV)	FWHM (mV)	Phonons	DFT (meV)	From reference (meV)
1	16.7 ± 0.1	11.6 ± 0.6	***Г***_4+_ (*h*-BN)/***Г***_6−_, ***Г***_2−_ (graphite)	11/14	16^19^/15^4^
2	33.5 ± 0.3	7.8 ± 1.0	***Г***_6−_, ***Г***_2−_ (graphene/*h*-BN hetero)	36	NA
3	46.4 ± 0.4	12.6 ± 1.7	***K***_6_ (graphene/*h*-BN hetero)	46	NA
4	66.7 ± 1.7	7.8 ± 6.9	***K***_6_ (graphite/graphene)	67	67^4^
5	83.8 ± 0.3	18.1 ± 1.5	***M***_2+_ (graphene/*h*-BN hetero)	86	NA
6	102.9 ± 0.3	9.4 ± 1.5	***Г***_3−_, ***Г***_4+_ (*h*-BN)/***Г***_4+_ (graphite)	99/110	102^19^ /102^4^
7	124.7 ± 2.4	19.7 ± 5.8	***K***_5_ (*h*-BN)/***K***_2_ (graphite)	129/124	133^21^/124^32^
8	134.8 ± 0.6	11.5 ± 2.5	***K***_5_ (graphene/*h*-BN hetero)	140	NA
9	156.4 ± 0.2	7.6 ± 0.7	***K***_1,2_ (*h*-BN)	155	158^19^
10	167.9 ± 0.1	12.6 ± 0.4	***Г***_5+_ (*h*-BN)	167	169^19^
11	186.3 ± 0.1	9.3 ± 0.4	LO overbending (*h*-BN)	184	185^19^
12	199.2 ± 0.2	7.1 ± 0.7	***Г***_5__+_/LO overbending (graphite)	198/202	200^32^

Position and FWHM for each peak are extracted from multi-peak Lorentzian fitting and the uncertainty represents one standard deviation. Listed are the most plausible phonons marked with red stars in Fig. 3 and DFPT-calculated energies at the high symmetry points in the Brillouin zone. Referenced energies for *h*-BN phonons are taken from Reich *et al*.^19^ and Kern *et al*.^21^, and those for graphite and graphene are from Mohr *et al*.^4^ and Vitali *et al*.^32^.
