# Supplementary material for: Vibrational Properties of h-BN and h-BN-Graphene Heterostructures Probed by Inelastic Electron Tunneling Spectroscopy
Source: Sci Rep. 2015 Nov 13;5:16642. doi: 10.1038/srep16642 (PMC4643226; doi:10.1038/srep16642)
Supplement: Supplementary Information [file srep16642-s1.pdf]

Supporting Online Material for

Vibrational Properties of *h*-BN and *h*-BN-Graphene Heterostructures Probed by Inelastic Electron Tunneling Spectroscopy

Suyong Jung*, Minkyu Park, Jaesung Park, Tae-Young Jeong, Hojong Kim, Kenji Watanabe, Takashi Taniguchi, Dong-Han Ha, Chanyong Hwang and Yong-Sung Kim*

* To whom correspondence should be addressed:
Email: syjung@kriss.re.kr (S. J.), yongsung.kim@kriss.re.kr (Y-S. K.)

This pdf file includes:

I. Phonon density-of-state analysis for *h*-BN and graphite/graphene
II. Phonon dispersions of graphene/*h*-BN heterostructure
III. References
IV. Figure S1 and S2

Supplementary Material

I. Phonon density-of-state analysis for *h*-BN and graphite/graphene

We calculate phonon dispersions of *h*-BN, graphite/graphene and graphene/*h*-BN heterostructures with density functional perturbation theory (DFPT)[1,2] implemented in the Vienna Ab initio Simulation Package (VASP)[3] within local density approximation (LDA)[4] as described in the Methods in the main manuscript. Figures S1a and S1d are the phonon dispersions of freestanding four-layer *h*-BN and graphite/graphene layers. We extract phonon density of states (DOS) of *h*-BN (Fig. S1b) and graphite/graphene (Fig. S1d) accounting for all the branches inside the Brillouin zone. As pointed out in the main manuscript, however, our experimental observations suggest that inelastic electron tunneling spectroscopy (IETS) signals from planar *h*-BN-based tunneling devices are not directly correlated with phonon density of states. For example, the most populated phonon modes of *h*-BN are related to $\boldsymbol{M}_{2+}$ and $\boldsymbol{M}_{3-}$ phonons (Fig. S1a and S1b), but the highest IETS signals are from $\boldsymbol{\Gamma}_{5+}$ phonon modes, which are rather well reproduced in phonon DOS plot, only following the branches connecting $\boldsymbol{\Gamma}$ and $\boldsymbol{K}$ high symmetry points, excluding the contribution from $\boldsymbol{M}$-point phonons (Fig. S1c). In addition, $\boldsymbol{M}_{3-}$ phonons have the highest phonon DOS among other graphite/graphene phonons (Fig. S1d and S1e), but the phonons at the symmetry point $\boldsymbol{\Gamma}$ are found to strongly interact with tunneling electrons, resulting in higher IETS signals (Fig. S1f and Fig. 2c).

II. Phonon dispersions of graphene/*h*-BN heterostructure

In our tunneling devices, single-layer graphene and thin *h*-BN are sequentially transferred on top of thick *h*-BN gate-insulating flake without additional efforts on the alignment of crystalline direction of graphene and either probe or bottom *h*-BN flake[5–7], which is confirmed

by the absence of additional electronic structures in tunneling spectra due to graphene/*h*-BN superlattice[8]. In forming graphene/*h*-BN heterostructures, graphene is aligned on top of *h*-BN layer in an either AA- or AB-stacking direction. In our tunneling junctions as wide as several microns, however, it is safe to assume that both AA- and AB-stacked graphene/*h*-BN junctions coexist. Thus, we should expect to measure phonon excitations from both AA- and AB-stacked graphene/*h*-BN junctions in our tunneling spectra.

As described in the main manuscript, we find that lattice motions of graphene and *h*-BN layers are influenced more when graphene/*h*-BN heterojunction is formed in an AA-stacked (Fig. S2a) than in an AB-stacked direction (Fig. S2b). For an AB-stacked heterojunction, we consider one carbon atom is placed directly on top of a boron atom and the other carbon atom is in the middle of a *h*-BN hexagon. We find the phonon dispersion of AB´-stacked heterojunction; one carbon atom is placed on top of a nitrogen atom and the other carbon atom is in the middle of a *h*-BN hexagon, is close to the dispersion of AB-stacked heterostructure, which is reported to be energetically more favorable to other AA- and AB´-stacked junctions[9].

Different from an AA-stacked graphene/*h*-BN heterostructure, low-energy out-of-plane motions of graphene and *h*-BN lattices are slightly modified when graphene is placed on top of *h*-BN in an AB-stacked direction , remaining the $\boldsymbol{K_6}$ phonon mode of *h*-BN close to the excitation of P_2 (Fig. S2b). In addition, $\boldsymbol{\Gamma_{6-}(\Gamma_{2-})}$ phonon mode, out-of-plane motions of graphene, is found to be blue-shifted at ≈ 6 meV much smaller than a ≈ 36 meV-shift for an AA-stacked case. The IETS signal relating to $\boldsymbol{\Gamma_{6-}(\Gamma_{2-})}$ phonon mode of an AB-stacked graphene/h-BN heterostructure is not observed in our data, probably due to weak IETS signals of the excitation at ≈ 6 meV in the proximity of the Fermi level (Fig. 2). Note that, however, $\boldsymbol{M_{2+}}$ and $\boldsymbol{K_5}$ phonon modes, which are ascribed to the IETS excitations at ≈ 84 meV (P_5) and ≈ 135 meV

(P_8), respectively, are found to be similar in both an AA- and an AB-stacked graphene/*h*-BN heterojunctions regardless of graphene-*h*-BN stacking order. For references, $\boldsymbol{M}_{2+}$ and $\boldsymbol{K}_5$ phonon modes of freestanding graphite were reported to form at ≈ 78 meV and ≈ 151 meV by inelastic x-ray scattering measruement[10].

References:

1. Gonze, X. & Lee, C. Dynamical matrices, Born effective charges, dielectric permittivity tensors, and interatomic force constants from density-functional perturbation theory. *Phys. Rev. B* **55,** 10355–10367 (1997).

2. Togo, A., Oba, F. & Tanaka, I. First-principles calculations of the ferroelastic transition between rutile-type and CaCl2-type SiO2 at high pressures. *Phys. Rev. B* **78,** 134106 (2008).

3. Kresse, G. & Hafner, J. Ab initio molecular dynamics for liquid metals. *Phys. Rev. B* **47,** 558 (1993).

4. Ceperley, D. M. & Alder, B. J. Ground state of the electron gas by a stochastic method. *Phys. Rev. Lett.* **45,** 566–569 (1980).

5. Hunt, B. *et al.* Massive Dirac fermions and Hofstadter butterfly in a van der Waals heterostructure. *Science* **340,** 1427–1430 (2013).

6. Dean, C. R. *et al.* Hofstadter's butterfly and the fractal quantum Hall effect in moiré superlattices. *Nature* **497,** 598–602 (2013).

7. Ponomarenko, L. *et al.* Cloning of Dirac fermions in graphene superlattices. *Nature* **497,** 594–597 (2013).

8. Moon, P. & Koshino, M. Electronic properties of graphene hexagonal boron nitride moiré superlattice. *Phys. Rev. B* **90,** 155406 (2014).

9. Slotman, G., de Wijs, G., Fasolino, A. & Katsnelson, M. Phonons and electron-phonon coupling in graphene-*h*-BN heterostructures. *Ann. Phys.* **526,** 381–386 (2014).

10. Mohr, M. *et al.* Phonon dispersion of graphite by inelastic x-ray scattering. *Phys. Rev. B* **76,** 035439 (2007).

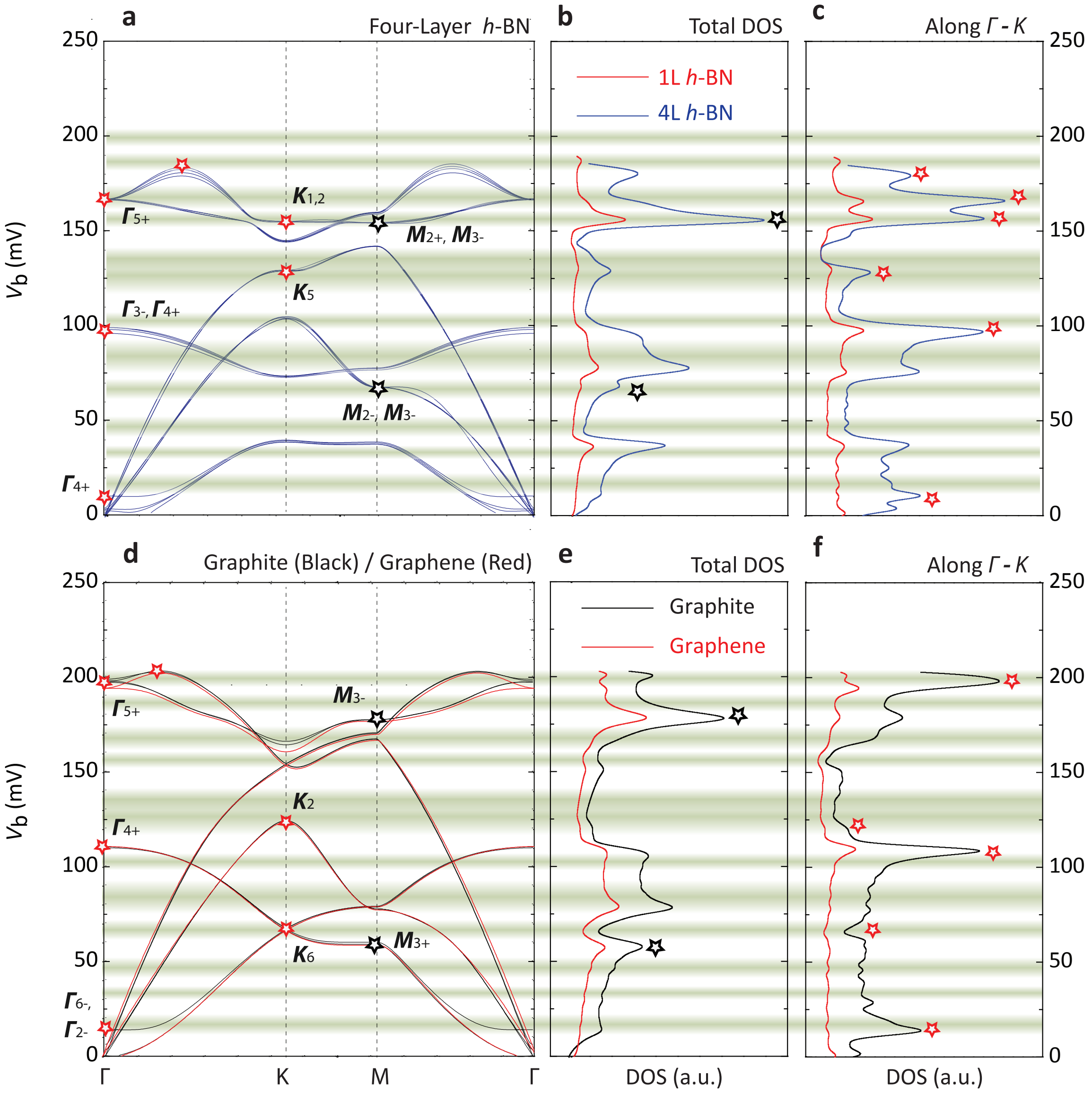

Figure S1: DFPT-calculated phonon dispersions of 4-layer *h*-BN (a) and graphite/graphene (d). Numerically obtained phonon DOS of single-layer (red)/four-layer (blue) *h*-BN (b) and graphite(black)/graphene(red) (e) inside the Brillouin zone, and those following the branches connecting high symmetry points ***Γ*** and ***K*** excluding the contributions from ***M***-point phonons (c, f). Black stars mark the ***M***-point phonons which have been missing in our data and red starts indicate the most plausible phonon modes.

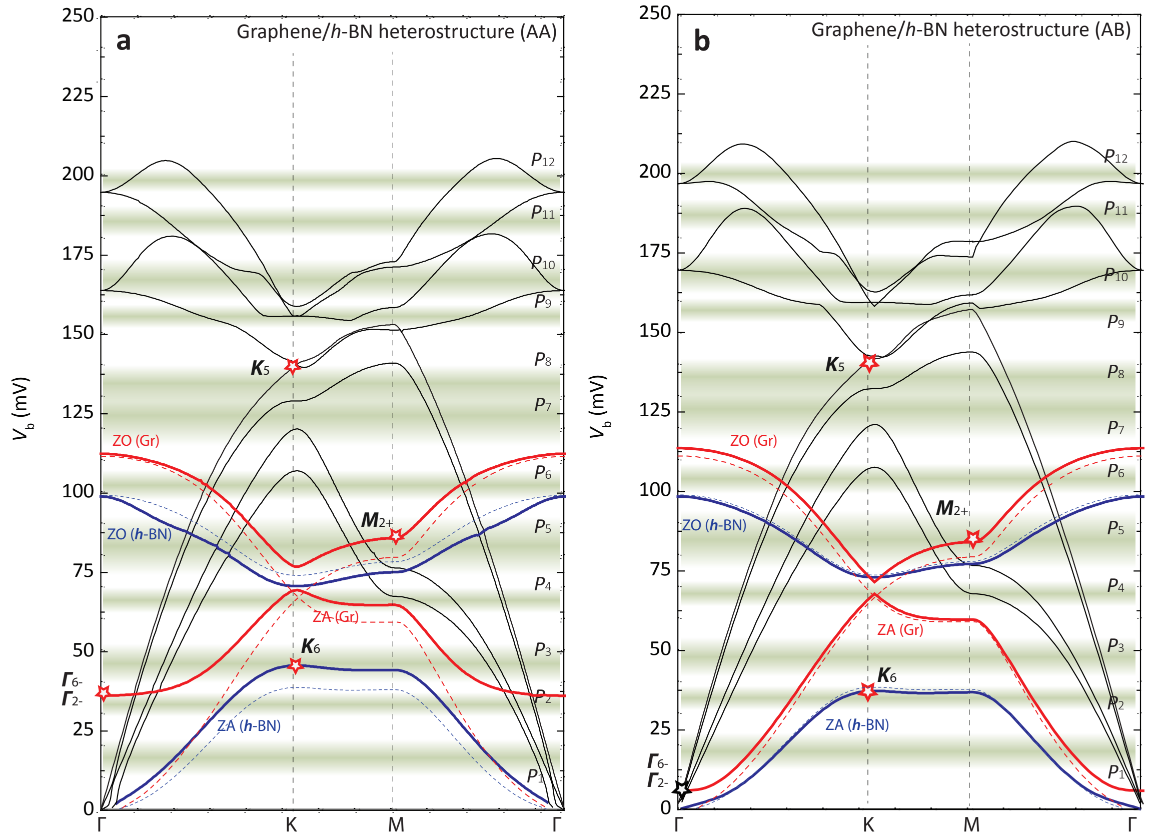

Figure S2: DFPT-calculated phonon dispersions of graphene/*h*-BN heterostructure stacked in the direction of AA (a) and AB (b). Out-of-plane lattice motions of *h*-BN and graphene are hardened for the case of AA-stacked heterostructure, contributing to the excitations of P_2, P_3 and P_5. Phonon dispersions of graphene and *h*-BN layers, however, are less affected when graphene/*h*-BN heterostructure is formed in the AB direction (b).
